# A new integrative approach to assess aortic stenosis burden and predict objective functional improvement after TAVR

**DOI:** 10.3389/fcvm.2023.1118409

**Published:** 2023-03-02

**Authors:** Jose M. de la Torre Hernandez, Gabriela Veiga Fernandez, Eyal Ben-Assa, Fermin Sainz Laso, Dae-Hyun Lee, Cristina Ruisanchez Villar, Piedad Lerena, Tamara Garcia Camarero, Jose M. Cuesta Cosgaya, Victor Fradejas-Sastre, Mercedes Benito, Sergio Barrera, Maria T. Garcia-Unzueta, Jonathan Brown, Aritz Gil Ongay, Javier Zueco, Jose A. Vazquez de Prada, Elazer R. Edelman

**Affiliations:** ^1^Division of Cardiology, Hospital Universitario Marqués de Valdecilla, Instituto de Investigación Valdecilla (IDIVAL), Santander, Spain; ^2^Department of Cardiology, Medical School, University of Cantabria, Santander, Spain; ^3^Division of Cardiology, Assuta Ashdod University Hospital, Ben-Gurion University of the Negev, Ashdod, Israel; ^4^Institute for Medical Engineering and Science, Massachusetts Institute of Technology, Cambridge, MA, United States; ^5^Análisis clínicos, Hospital Universitario Marqués de Valdecilla, Instituto de Investigación Valdecilla (IDIVAL), Santander, Spain; ^6^Cardiovascular Division, Harvard Medical School, Brigham and Women’s Hospital, Boston, MA, United States

**Keywords:** aortic stenosis, arterial pulse wave, transcatheter aortic valve replacement, functional recovery, clinical outcomes

## Abstract

**Background:**

A non-negligible rate of patients undergoing transcatheter aortic valve replacement (TAVR) do not report symptomatic improvement or even die in the short-midterm. We sought to assess the degree of objective functional recovery after TAVR and its prognostic implications and to develop a predictive model.

**Methods:**

In a cohort of patients undergoing TAVR, a prospective evaluation of clinical, anatomical, and physiological parameters was conducted before and after the procedure. These parameters were derived from echocardiography, non-invasive analysis of arterial pulse waves, and cardiac tomography. Objective functional improvement 6 months after TAVR was assessed using a 6-min walk test and nitro-terminal pro-brain natriuretic peptide (NT-proBNP) levels. The derived predictive model was prospectively validated in a different cohort. A clinical follow-up was conducted at 2 years.

**Results:**

Among the 212 patients included, objective functional improvement was observed in 169 patients (80%) and subjective improvement in 187 (88%). Patients with objective functional improvement showed a much lower death rate at 2 years (9% vs. 31% *p* = 0.0002). Independent predictors of improvement were as follows: mean aortic gradient of ≥40 mmHg, augmentation index_75_ of ≥45%, the posterior wall thickness of ≤12 mm, and absence of atrial fibrillation. A simple integer-based point score was developed (GAPA score), which showed an area under the curve of 0.81 for the overall cohort and 0.78 for the low-gradient subgroup. In a validation cohort of 216 patients, these values were 0.75 and 0.76, respectively.

**Conclusion:**

A total of 80% of patients experienced objective functional improvement after TAVR, showing a significantly lower 2-year mortality rate. A predictive score was built that showed a good discriminative performance in overall and low-gradient populations.

## Introduction

Transcatheter aortic valve replacement (TAVR) improves survival and quality of life in the majority of patients with severe aortic stenosis (AS). Nonetheless, up to one-fifth of patients continue to have a poor quality of life after TAVR, with an additional similar proportion not surviving 1 year after the procedure (1). However, given the very poor prognosis associated with non-procedural management of symptomatic severe AS (2), the decision will usually be made to proceed with TAVR even if there is a concern for a sub-optimal result.

Nonetheless, it is important to explore and understand the factors associated with a sub-optimal outcome to inform decisions regarding the optimal timing of TAVR and/or adjunctive interventions that may improve outcomes after TAVR such as particular medications and rehabilitation.

Furthermore, the identification of such predictors is important to help in the decision-making when the indication for TAVR is doubtful because of the risk of futility due to frailty or relevant comorbidities, certain cases of low-flow/low-gradient AS, and finally, the unclear symptomatic status associated with moderate stenosis (3, 4).

Increasingly, we appreciate that AS really represents a complex, multifaceted set of syndromes that may present in a range of manners and is not isolated to calcific degeneration of the aortic valve alone. Systemic perfusion and ventricular work can more fully define the AS state and the response to TAVR than the valve gradient alone (5–8). Thus, indices of the functional state preprocedure and improvement post-TAVR should include more than the valve area estimation alone. With such a more inclusive perspective, the indication for and timing of TAVR could be enhanced, adding precision to the decision-making process.

The objective of the present study was to establish the degree of objective functional recovery of the patient after TAVR; to evaluate its prognostic effects; to identify the baseline clinical, physiological, and anatomical variables independently associated with this improvement; and to develop a predictive model for response to intervention.

## Materials and methods

### Population

During the period from February 2018 to June 2020, all patients scheduled for TAVR who met the following criteria were included in the study: (1) diagnosis of significant and symptomatic AS, (2) indication for TAVR established by the institutional heart team, and (3) undergoing a TAVR procedure through femoral artery access. Patients who did not consent or who exhibited cognitive impairment that prevented them from properly understanding the investigational procedures were excluded. The predictive model was derived from this cohort and validated in a separate sample of patients selected under the same criteria, recruited during a consecutive period, from July 2020 to March 2022.

All the procedures were performed in the appropriate setting of a catheterization laboratory dedicated to structural heart interventions. The local TAVR program was started in 2009 and is based on balloon-expandable prosthetic valves. The study was approved by the corresponding Institutional Review Board and all participating patients signed the informed consent after a proper explanation of the investigational procedures. The database was completely anonymized.

### Pre-procedural and post-procedural clinical and functional evaluation

The workflow of the study is shown in Supplementary Figure 1. Once the patients received the indication for TAVR from the Heart Team, they were evaluated in the outpatient office for structural heart interventions. In this visit, all the clinical information was collected, and the functional status of the patient including quality of life and frailty was assessed using accepted questionnaires [SF 36, EQ 5D, Barthel I, essential frailty tool set, NYHA class, and the Kansas Cardiomyopathy Questionnaire (KCCQ)], a 6-min walk test, and the determination of nitro-terminal pro-brain natriuretic peptide (NT-proBNP) levels in the baseline condition.

On the same day of the procedure, just before entering the Cath Lab, all patients underwent simultaneous examination of hemodynamics and cardiac imaging. The former was determined with a non-invasive method that provides central pressure from peripheral measurements, by the SphygmoCor XCEL device (AtCor Medical, Naperville, IL, USA). Our group has previously confirmed a strong correlation between these non-invasive metrics and invasive hemodynamic measures (9). In this study, we observed a certain degree of underestimation of aortic systolic blood pressure by the non-invasive method, being this tendency is more pronounced after TAVR when systolic blood pressure is higher. The reported better correlation with central invasive pressure shown for the estimated central pressure with respect to the cuff-brachial systolic blood pressure before TAVR would make it the best method to be used in the calculation of non-invasive metrics related to the valvulo-arterial load in patients with AS. Other groups have also used non-invasive pressure (from carotid or derived radial applanation tonometry) in the setting of AS (10–12). Three repeated measurements were performed and averaged to calculate the non-invasively measured central pressure values, amplification phenomenon, as the difference between systolic blood pressure values measured in the brachial artery with respect to systolic blood pressure values in the ascending aorta, and augmentation index_75_ (AIx_75_), as the percentage increase in blood pressure from augmentation of pulse pressure by the reflected wave on the forward wave standardized to a heart rate of 75 bpm. All measurements were taken by well-trained personnel with experience in the setting of a hypertension clinic. Protocol-specific transthoracic echocardiography examination was also performed to obtain a complete set of data addressing morphological and functional aspects of the aortic valve, the left ventricle, and the ascending aorta.

In the validation cohort, the same baseline echocardiographic and SphygmoCor XCEL examination were performed, and at 6 months follow-up, the objective functional status of patients was assessed by the 6-min walk test and NT-proBNP levels.

### Physiological and anatomical variables analyzed

All clinical and functional assessment was repeated 30 days, 6 months, and 12 months after the TAVR procedure by the same team and in the same setting (Supplementary Figure 2).

### Endpoints and definitions

The primary endpoint of the study was to determine the rate of objective functional improvement of the patient with AS after the TAVR procedure. This was defined as the achievement at 6 months of an increase of at least 10% in the distance covered during the 6-min walk test, or a reduction of at least 50% in NT-proBNP blood levels with respect to pre-TAVR when this 10% increase was not evident. In this way, with both criteria, the potential presence of factors that limit the speed of gait and that are not related to cardiovascular capacity were taken into consideration. In those few cases in which the patient was in such a compromised functional situation that he was unable to perform the walk test, improvement was estimated positive when the patient survived the procedure and was able to perform a 6-min walk test at follow-up, showing a corresponding reduction in NT-proBNP levels with respect to the pre-TAVR condition.

For those patients who died before the 6 months landmark and after the 30-day evaluation improvement was based on this earlier evaluation, but this was considered negative if patients suffered or died of heart failure afterward. For those who died before the 30-day follow-up, those who died because of heart failure were considered without improvement and the rest who died from other causes were excluded from the analysis since no functional evaluation was available.

Thus, two subgroups were defined, one subgroup showing objective functional improvement (FUNC+) and the other without objective functional improvement (FUNC−).

Subjective improvement was considered if patients reported a positive change in at least one class of the NYHA classification and/or an increase in at least 10 points in the KCCQ. All patients underwent systematic clinical follow-up at 2 years using standardized endpoints definitions for transcatheter aortic valve implantation as defined in the VARC-2 consensus document (13).

### Statistical analysis

Continuous variables are presented as mean ± standard deviation or median (interquartile range) according to the type of distribution, and categorical variables as percentages. Distribution was assessed for each variable with the Shapiro–Wilk test. Accordingly, continuous variables were compared with the Student’s *t*-test if they followed a normal distribution and by non-parametric tests when this was not the case. The categorical variables were compared with the chi-square test or the Fisher exact test, as required.

Multivariable logistic regression analysis identified independent predictors of objective functional improvement post-TAVR. Covariates that showed a univariate relationship with the outcome (*p* < 0.2) were entered into the multivariable logistic regression model. Then, stepwise elimination analysis was performed to define a useful subset of predictors. The risk score was then constructed to predict functional improvement post-TAVR using a regression coefficient-based scoring method. A simple integer-based point score was obtained for each predictive variable, dividing each *b* coefficient by the absolute value of the smallest coefficient, multiplied by 5, and rounded to the nearest integer. The discriminating power of the score was evaluated by the area under the curve from the receiver operating characteristic (ROC) analysis. The adequacy of the model was checked using the Hosmer–Lemeshow goodness-of-fit test. Box-and-whisker plots were built to show the baseline and post-TAVR evolution of variables according to improvement subgroups. Kaplan–Meier curves for event-free survival were obtained for each group and compared using the log-rank test and the hazard ratios with a 95% confidence interval. Values of *p* < 0.05 were considered statistically significant. The statistical packages SPSS 25.0 and Medcalc 20.009 were used throughout.

## Results

During the period from February 2018 to June 2020, a total of 212 patients were included in the derivation cohort, and 216 patients as part of the validation cohort in the consecutive period from July 2020 to March 2022 (Supplementary Figure 3). The clinical characteristics of the patients and the procedural features in the derivation cohort are shown in Supplementary Table 1. In-hospital death occurred in four (1.9%). In the remaining 208 patients, TAVR indeed induced a range of changes in patho-physiological parameters from baseline at 6 months post-TAVR (Supplementary Table 2) and clinical outcomes at 2 years follow-up after discharge (Supplementary Table 3).

**FIGURE 1:**
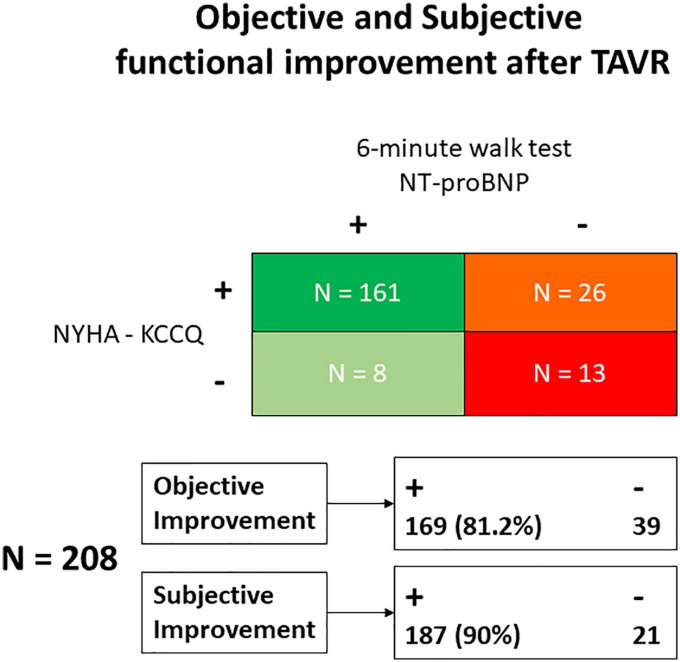
Classification of patients according to the objective and subjective (self-reported) functional improvement observed after TAVR. KCCQ, Kansas City Cardiomyopathy Questionnaire; NT-proBNP, Nitro-terminal-pro brain natriuretic peptide; NYHA, New York Heart Association; TAVR, Transcatheter Aortic Valve Replacement.

**FIGURE 2:**
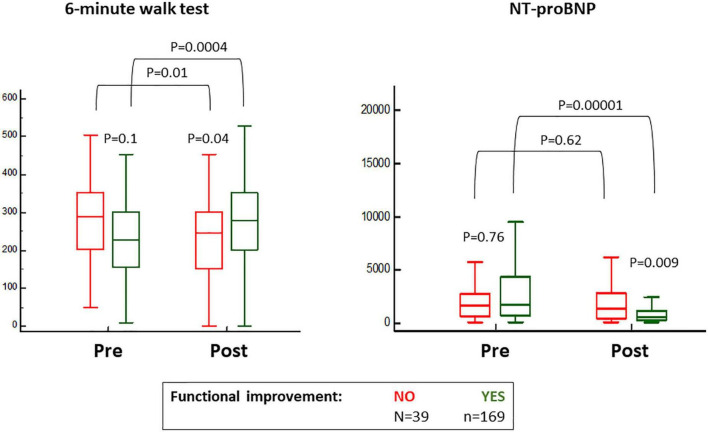
Baseline and 6 months post-transcatheter aortic valve replacement (TAVR) values for the 6-min walk test and blood levels of nitro-terminal pro-brain natriuretic peptide (NT-proBNP), according to the classification for objective functional improvement.

While 187 (88%) patients indicated subjective amelioration of symptoms, 169 (80%) projected objective findings to suggest an improvement (Figure 1). By definition, NT-proBNP was significantly reduced and the 6-min walk improved in the group with benefit and not in the group without functional improvement where there was even reduction in distance covered during the 6-min walk test (Figure 2). These trends were equally evident in the 26 patients with subjective but not an objective improvement as they demonstrated a significant decrease in the 6-min walk test distance, from 293 ± 100 to 237 ± 116 meters (*p* < 0.01) with no changes in NT-proBNP levels, from 1,298 (350–2,175) to 1,118 (320–1,895) (*p* = 0.2).

**FIGURE 3:**
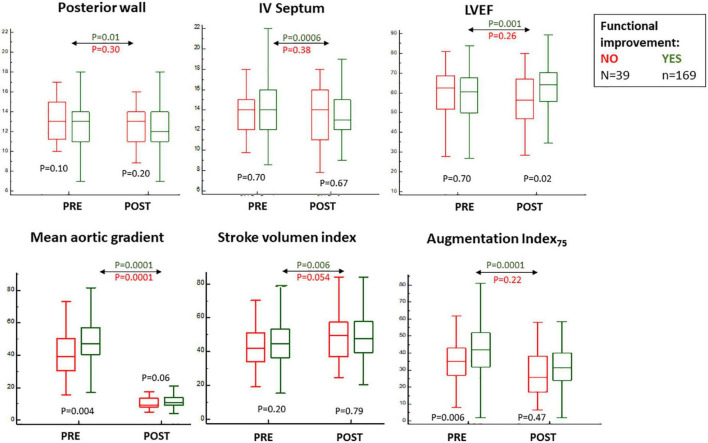
Baseline and 6 months post-transcatheter aortic valve replacement (TAVR) values for echocardiographic parameters and augmentation index_75_ measurements according to the classification for objective functional improvement.

Among all clinical variables, only atrial fibrillation stood out between groups—less evident in those with (FUNC+) as opposed to those without (FUNC−) objective functional improvement (Table 1). No differences were found in procedural data, except for a higher rate of post-TAVR stroke in the FUNC− group (Table 1), and at baseline, the FUNC+ showed higher aortic gradients, lower central pressures, and higher AIx_75_ but similar LVEF, stroke volume index, and myocardial wall thickness (Figure 3 and Table 2). Comparing the pre- to post-TAVR changes, in the FUNC+ group, a significant change was observed in LVEF, septal and posterior wall thickness, SBP, DBP, and AIx_75_ (Figure 3 and Table 2). The AIx_75_ was higher at baseline and decreased significantly in the FUNC+, whereas the pulse wave velocity, being comparable at baseline and after TAVR, clearly goes up in both groups.

**TABLE 1:** Baseline and procedural characteristics according to objective functional improvement.

	Functional improvement *N* = 169	Not functional improvement *N* = 39	*P*-value
Age, years	81.3 (77.2–84.3)	83.7 (75.8–87)	0.16
Female gender	95 (56.2%)	20 (51.3%)	0.59
Diabetes	50 (29.6%)	16 (41%)	0.18
High blood pressure	142 (84%)	31 (79.5%)	0.49
Dyslipidemia	123 (72.8%)	30 (76.9%)	0.69
Coronary artery disease	50 (29.6%)	15 (38.5%)	0.34
Previous MI	15 (8.9%)	2 (5.1%)	0.75
Previous PCI	32 (18.9%)	8 (20.5%)	0.82
<6 months	17 (10.1%)	3 (7.7%)	1
Previous CABG	4 (2.4%)	0	1
Carotid disease	3 (1.8%)	2 (5.1%)	0.24
Peripheral vascular disease	14 (8.3%)	3 (7.7%)	1
Mitral valve disease	29 (17.2%)	6 (15.4%)	1
Atrial fibrillation	53 (31.4%)	22 (56.4%)	0.005
Previous pacemaker	6 (3.6%)	4 (10.3%)	0.16
GFR < 60 ml/min	69 (40.8%)	22 (56.4%)	0.11
GFR < 30 ml/min	8 (4.7%)	3 (7.7%)	0.44
Pulmonary disease	26 (15.4%)	7 (17.9%)	0.64
Liver disease	5 (3%)	3 (7.7%)	0.17
History of cancer	32 (18.9%)	8 (20.5%)	0.82
Agatston calcium score	2981.5	2725	0.82
	(1,861–3,723.5)	(1,625–4877.5)	
EuroSCORE II	3.3 (2.6)	3.3 (2.9)	0.95
STS-score mortality	2.8 (2–4)	3.2 (2.4–4.4)	0.21
**Symptomatic and functional status**			
6 min walking test (m)	236 ± 107.1	273 ± 110	0.12
NT-proBNP (pg/ml)	1,772 (727–4,263.5)	1,718 (667–3,156)	0.45
NYHA			0.12
Class I	0	0	
Class II	105 (62.1%)	31 (79.5%)	
Class III	58 (34.3%)	8 (20.5%)	
Class IV	6 (3.6%)	0	
KCCQ	59.3 ± 13.4	65.3 ± 12.9	0.03
Test SF-36	41 (31–56)	46 (33–63)	0.34
Test EQ-5D	50 (50–60)	50 (45–70)	0.98
Barthel index	100 (95–100)	100 (100–100)	0.15
Charlson comorbidity index	5 (4–6)	5 (4–8)	0.34
Essential Frailty Toolset			0.20
0	52 (30.8%)	18 (46.2%)	
1–2	89 (52.7%)	19 (48.7%)	
3–4	28 (16.5%)	2 (5.1%)	
5	0	0	
**Procedural characteristics**			
Size of valve prothesis			0.30
23 mm	70 (41.4%)	11 (28.2%)	
26 mm	80 (47.3%)	23 (59%)	
29 mm	18 (10.7%)	5 (12.8%)	
Predilatation	76 (45%)	20 (51.3%)	0.48
Postdilatation	7 (4.1%)	3 (7.7%)	0.42
Coronary obstruction	2 (1.2%)	1 (2.6%)	0.47
Valve embolization	0	0	
Second valve implantation	0	0	
Aortic annulus rupture	0	0	
Stroke	0	1 (2.5%)	0.04

Values are *n* (%), mean ± SD, or median (25–75th interquartile range), depending on the variable distribution. MI, Myocardial infarction; PCI, Percutaneous coronary intervention; CABG, Coronary artery bypass graft; GFR, Glomerular filtration rate; STS-score, Society of Thoracic Surgeons score; NYHA, New York Heart Association; KCCQ, Kansas City Cardiomiopathy Questionnarie; EQ-5D, European Quality of life 5 Dimensions.

**TABLE 2:** Physiological and anatomical variables in relationship with objective functional improvement.

	Pre-TAVR			Post-TAVR			Pre- vs. Post-TAVR	
	**Functional improvement** ***N* = 169**	**Not functional improvement** ***N* = 39**	**P-value**	**Functional improvement** ***N* = 169**	**Not functional improvement** ***N* = 39**	***P*-value**	**Functional improvement** ***P*-value**	**Not functional improvement** ***P*-value**
Left ventricular ejection fraction (%)	60 (50–67)	62 (51–69)	0.70	64 (55–70)	56 (47–67)	0.02	0.001	0.26
Stroke volume index (ml/m^2^)	45.5 ± 14	42.9 ± 15.4	0.20	48.8 ± 13	48.8 ± 15.3	0.79	0.006	0.054
Interventricular septum (mm)	14.2 ± 2.5	14 ± 2.7	0.70	13.4 ± 2.2	13.7 ± 2.8	0.67	0.0006	0.38
Posterior wall (mm)	12.7 ± 2.3	13.3 ± 2.6	0.10	12.1 ± 2.2	12.8 ± 2.3	0.20	0.01	0.30
Maximal aortic gradient (mmHg)	82 (70–97.4)	65.6 (55–83.2)	0.003	22 (17.5–29)	18.5 (15.7–25)	0.03	0.0001	0.0001
Mean aortic gradient (mmHg)	47 (40.5–57)	39.4 (30.3–51)	0.004	11 (9–14)	9.5 (8–13.5)	0.06	0.0001	0.0001
Aortic valve área (cm^2^)	0.7 (0.57–0.86)	0.73 (0.6–0.94)	0.23	1.8 (1.5–2.3)	2.1 (1.8–2.4)	0.052	0.0001	0.0001
Indexed aortic valve área (cm^2^/m^2^)	0.4 (0.32–0.5)	0.42 (0.34–0.54)	0.26	1 (0.9–1.22)	1.2 (0.9–1.5)	0.06	0.0001	0.0001
Energy loss index (cm^2^/m^2^)	0.44 (0.35–0.5)	0.48 (0.4–0.63)	0.29	1.5 (1.14–2)	1.5 (0.9–2.2)	0.96	0.0001	0.0001
LVOT velocity/Aortic valve velocity	0.2 (0.16–0.25)	0.23 (0.19–0.28)	0.09	0.5 (0.42–0.6)	0.56 (0.4–0.64)	0.23	0.0001	0.0001
**Sphygmocor XCEL**								
Central SBP (mmHg)	130 ± 19.2	136.5 ± 20.7	0.04	139 ± 19	136 ± 21.5	0.44	0.0001	0.75
Central DBP (mmHg)	72 (65–80)	80 (68–90)	0.01	80 (72–86)	77 (70–89)	0.66	0.0001	0.87
Central PP	56.8 ± 17.6	57.8 ± 15.7	0.34	59.3 ± 15.4	57 ± 15.6	0.34	0.046	0.001
Pulse wave velocity (m/s)	11.4 ± 3.2	12.6 ± 3	0.20	14.3 ± 3.3	15 ± 3	0.70	0.0001	0.0004
Amplification phenomenon	24 (18–32)	22 (14–29)	0.15	20 (14–28)	19 (10–24)	0.15	0.0001	0.07
Augmentation index_75_	41.7 ± 16.2	32.6 ± 14.5	0.006	31 ± 12.6	29 ± 13	0.47	0.0001	0.22
Systemic vascular resistance (dyna.seg.cm^–5^)	1,376.5 (1,051–1704.5)	1,362 (1,029–1,793)	0.77	1,380 (1,062–1,721)	1,347.5 (1,002–1,684)	0.64	0.70	0.14
**Aortic distensibility (cm^2^ dyna^–1^ 10^–6^)**								
TTE	1 ± 1.2	1.5 ± 1.2	0.07	0.9 ± 0.9	1.3 ± 1.3	0.01	0.09	0.62
TEE	1.2 ± 0.9	1.5 ± 1.2	0.26	1.2 ± 1	1 ± 0.9	0.31	0.72	0.17
Valvuloarterial impedance (Zva) (mmHg.ml^–1^.m^–2^)	4.1 (3.3–5.1)	4.2 (3.6–5.7)	0.22	3.2 (2.63–3.9)	3.2 (2.6–4.3)	0.98	0.0001	0.0001
**Functional status assessment**								
NT-proBNP (pg/ml)	1,772 (727–4,263.5)	1,718 (667–3,156)	0.74	524 (236–1,195.8)	1,371.5 (425.8–2,906.5)	0.009	0.0001	0.62
6 min walking test (m)	236 ± 107.1	273 ± 110	0.13	279.2 ± 110.7	237.1 ± 120	0.04	0.0004	0.01

Values are *n* (%), mean ± SD, or median (25–75th interquartile range), depending on the variable distribution. TAVR, Transcatheter aortic valve replacement; SVi, stroke volume index; LVOT, Left ventricular outflow tract; SBP, Systolic blood pressure; DBP, Diastolic blood pressure; PP, Pulse pressure; TTE, Transthoracic echocardiogram; TEE, Transesophageal echocardiogram; Zva, Valvuloarterial impedance; NT-proBNP, N-terminal-pro hormone brain natriuretic peptide.

**FIGURE 4:**
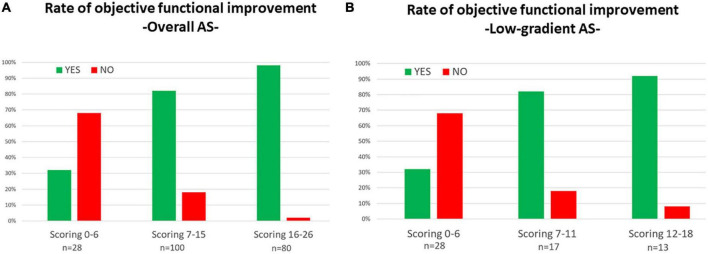
Rates for objective functional improvement after transcatheter aortic valve replacement (TAVR) according to different ranges of values for the GAPA Score (mean gradient, augmentation index_75_, posterior wall diastolic thickness, and atrial fibrillation) in all populations with aortic steno **(A)** and the low-gradient aortic stenosis subpopulation **(B)**.

The independent baseline predictors of functional improvement were mean aortic gradient of ≥40 mmHg, AIx_75_ of ≥45%, posterior wall diastolic thickness of ≤12 mm, and the absence of atrial fibrillation (Table 3). A score was built, the GAPA score, whose discriminative performance yielded an AUC of 0.81 for the overall cohort, 0.78 for the low-gradient AS subgroup, and 0.77 for the low-gradient/low-flow AS subgroup (Table 3 and Supplementary Figure 4). The best cutoff values were 8, 6, and 6. This score performed better than classic parameters related to AS (Supplementary Table 4). The rates of objective functional improvement for different ranges of the GAPA score values in the global and low-gradient AS populations are represented in Figure 4.

**TABLE 3:** Analysis for baseline predictors of objective functional improvement.

Independent anatomic-physiological predictors:		
	**OR 95% CI**	***p***
Mean aortic gradient	1.05 (1.01–1.08)	0.01
Augmentation index_75_	1.03 (1.008–1. 06)	0.01
Posterior wall thickness	0.81 (0.70–0.98)	0.02
**Added clinical variables in the regression model (showing *p* < 0.2 in univariant analysis) and; Anatomic-physiological predictors entered with the best cutoff value in ROC**		
Mean aortic gradient ≥ 40 mmHg	4.3 (2–10)	0.001
Augmentation index_75_ ≥ 45%	3.8 (1.4–10.3)	0.007
Posterior wall thickness ≤ 12 mm	3 (1.3–6.9)	0.018
No atrial fibrillation	2.5 (1.1–5.5)	0.02
**To generate a simple integer-based point score for each predictive variable, each beta coefficient was divided by the absolute value of the smallest coefficient, multiplied by 5, and rounded to the nearest integer.**		
	**GAPA score**	
**General AS Score**		
Mean aortic gradient ≥ 40 mmHg	8	
Augmentation index_75_ ≥ 45%	7	
Posterior wall thickness ≤ 12 mm	6	
No atrial fibrillation	5	
	AUC 0.81	*p* < 0.001
**Low-Gradient AS score**		
Augmentation index_75_ ≥ 45%	7	
Posterior wall thickness ≤ 12 mm	6	
No atrial fibrillation	5	
	AUC 0.78	*p* < 0.001

The GAPA score development.

In Supplementary Figure 5, the scatter plot is shown for the baseline values of mean aortic gradient and AIx75, indicating those associated with objective functional improvement or not. In patients showing measurements of both parameters under the respective cutoff values (40 mmHg and 45%, respectively), the rate of functional improvement was just 52%.

**FIGURE 5:**
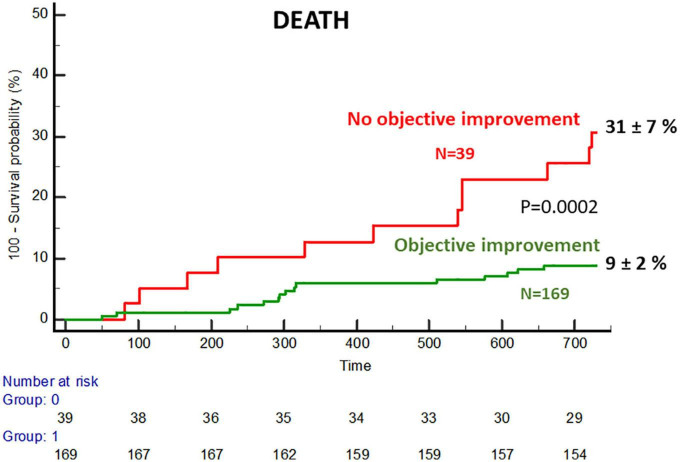
Cumulative incidence of mortality in the subgroups with and without objective functional improvement.

The FUNC- group showed a significantly higher rate of death and admissions due to heart failure at 2 years (Figure 5 and Supplementary Table 5). Based on objective and subjective functional improvement, four subgroups were identified with 2-year death rates, as shown in Supplementary Table 6.

The GAPA score was then applied to a validation cohort of 206 patients. The clinical and procedural characteristics of the validation cohort are described in Supplementary Tables 7, 8. Applying the same definition for functional improvement in the 216 patients examined at 6 months follow-up, the GAPA score showed an AUC of 0.75 for the total cohort and 0.76 for the low-gradient subgroup (both *p* < 0.001).

In this study, we have focused on the prediction of improvement before TAVR and, therefore, only baseline variables were analyzed for this purpose. Of course, post-TAVR early outcomes such as new atrial fibrillation and paravalvular aortic regurgitation could influence improvement but would not be useful for pre-procedure estimation.

In this regard, new atrial fibrillation after TAVR was observed in 16/169 (9.4%) of patients improving and 4/39 (10.2%) of those not improving. Thus, it seems not to have the clear influence that was found for the presence of atrial fibrillation at baseline. Regarding residual aortic regurgitation, all these patients were treated with the balloon-expandable prosthesis in which the rate of paravalvular leak is lower. Nonetheless, at 6 months, 13% of those who did not improve and 9% of those who improved had more than mild paravalvular regurgitation (*p* = 0.4), though numbers do not provide enough statistical power.

## Discussion

Aortic stenosis is indeed a complex syndrome that can indeed benefit from TAVR. A careful 2-year follow-up of hemodynamics and outcomes allow us to conclude that (1) objective functional improvement of the patients post-intervention is high but lower than that reported subjectively by the patients; (2) the TAVR procedure induces a series of anatomical-physiological changes apart from those related to aortic valve function, with affects on cardiac performance and afterload reduction, whose magnitude differs with objective functional improvement; and (3) it is possible to identify a series of predictive parameters for objective functional improvement in the pre-TAVR phase and thus design a score based on mean aortic gradient, AIx_75_, posterior wall thickness, and absence/presence of atrial fibrillation, whose diagnostic performance was good both in the global and low gradient populations of derivation and validation cohorts.

A certain proportion of patients who undergo TAVR do not report any symptomatic improvement or even die in the short to medium term after the procedure (1). In addition, functional improvement is usually based on questionnaires reported by the patient, which introduces a certain subjective component. On the other hand, in some patients, the indication for TAVR is doubtful due to an ambiguous assessment of aortic stenosis (i.e., low gradient), the presence of co-morbidities that do not allow estimating the symptomatic nature of aortic stenosis, or portend a certain adverse prognosis or because borderline values in frailty tests conducted to assess the potential futility of the procedure.

In addition, it is crucial to identify patients at risk for a sub-optimal outcome after TAVR in order to apply adjunctive interventions that may improve outcomes after TAVR, such as specific medications and rehabilitation.

In our study, about 20% of the patients did not show objective functional improvement, although more than half of them self-reported some improvement. In the latter, a poor performance was even observed in the 6-min walk test with no change in NT-proBNP levels. Remarkably, we were also able to verify that the absence of objective functional improvement was associated with a much worse prognosis in terms of mortality.

### The predictive model of objective functional improvement after TAVR

Of the large number of variables considered in our comprehensive, multimodal prospective assessment statistical analysis identified the mean aortic gradient, augmentation index, diastolic thickness of the posterior myocardial wall, and atrial fibrillation as independent baseline predictors of objective functional improvement after TAVR.

It is increasingly clear that atrial fibrillation is associated with negative outcomes after TAVR. All-cause mortality, rehospitalization, and advanced heart failure symptoms are more common in patients with atrial fibrillation (14, 15). Atrial fibrillation could be indicating a more advanced degree of heart damage derived from aortic stenosis and other frequently associated pathologies such as hypertension, ischemic heart disease, and dilated cardiomyopathy.

The AIx is defined according to the inflection point observed on the upstroke of the pressure waveform, as the marker of the arrival of the backward reflective wave and its superposition with the forward wave. Thus, AIx is often considered a vascular measure of aortic stiffness and wave reflection (16). Nonetheless, there is meaningful evidence that suggests that the AIx might not be a marker for aortic stiffenings, such as the nonlinear relationship between this parameter and age as well as the association between lower AIx and a higher burden of cardiovascular risk (17, 18).

With regards to the AIx in the setting of aortic stenosis, this has been identified as an independent predictor of mortality in a cohort of 133 patients with moderate to severe isolated AS and preserved LV ejection fraction (19). At the same time, another previous study, using the invasive methodology, showed a decrease in AIx after TAVR (42 ± 12% to 19 ± 11%; *p* < 0.001) despite an unaffected arterial compliance and reflection coefficient, which seriously challenges the view of AIx as a marker of stiffness in patients with AS (20). This finding was also reported in other investigations of pressure waveforms (21). In a study including 88 patients with severe AS undergoing intervention with TAVR or surgical aortic valve replacement, the AIx was somehow related to a poorer symptomatic recovery; however, the small sample size, the non-consistent findings across the complete analysis, and the statistical approach make these results questionable (12).

Our study and others suggest then that the decrease in AIx reflects changes in the ventricular-aortic interaction due to the resolution of AS (20). A plausible explanation for the decrease in AIx is that it is associated with the timing/slope of the enhanced forward wave. An earlier and steeper increase in early systolic (forward mainly) waves will result in much higher pressures before the arrival of the reflected wave, driving a smaller contribution of wave reflections to the total pulse pressure and a lower AIx (20). This explanation serves as well to support the finding of a higher AIx at baseline in patients with aortic stenosis as a predictor of objective functional recovery.

A less than 13 mm posterior wall thickness was an independent predictor for objective functional improvement. A reduced diastolic wall thinning indicates decreased LV compliance and distensibility in keeping with linear elastic theory (22). An echocardiographic parameter derived from the posterior wall measurements, the diastolic wall strain is defined as (LVPWs-LVPWd)/LVPWs, where LVPWs, left ventricular posterior wall thickness in systole and LVPWd, left ventricular wall thickness in diástole, is a novel method described as non-invasive and easily reproducible preload independent estimator of LV stiffness (22, 23). The thicker the posterior wall in diastole, the lower the DWS, which has been shown previously to be associated with poor prognosis in patients with heart failure with preserved ejection fraction (22), and in patients with AS (24). All these findings support the prognostic value we have found for the baseline posterior wall thickness in patients with AS undergoing TAVR.

Other studies have also aimed to establish predictors of functional improvement after TAVR. In a study in which our group participated, relatively complex physiological parameters such as left ventricular stroke work and vascular impedance spectrums in the frequency domain were identified as predictors of a better quality of life 1 month after the procedure, highlighting the importance of a comprehensive assessment of the ventriculo-arterial physiology (8).

On the contrary, in a much more clinical setting, a model has been developed and externally validated to estimate the risk of a poor outcome (using a composite of death or self-reported poor quality of life) at 1 year among high-risk and inoperable patients who underwent TAVR as part of pivotal trials (25, 26). This model, which consists of six variables, has recently been tested in a larger, more contemporary TAVR population, but did not calibrate well (1). After re-estimating the intercept and coefficients, it performed better with a C index of 0.65.

The model developed in our study comes from the integration of multiple clinical, anatomical, and physiological variables, and seems to perform both in a contemporary population undergoing TAVR, as well as in the subgroup of patients with low-gradient AS regardless of stroke volume.

### Performance of the model in low-gradient AS

The heterogenous low-gradient AS condition appears to have a poor prognosis and requires prompt assessment and intervention. Patients with low-gradient/low-flow AS, particularly with low ejection fraction, have significantly worse medium-term to long-term survival compared with all other patients following TAVR (27). In this setting, a low-dose dobutamine stress echocardiography is recommended to distinguish between true severe and pseudo-severe aortic stenosis. However, after TAVR, the absence of contractile reserve at baseline in this test was not associated with any negative effect on clinical outcomes or LVEF changes at follow-up (27).

The scenario of low-flow/low-gradient AS with preserved ejection fraction may also result from conditions associated with low stroke volume and requires careful exclusion of measurement errors and other explanations for the echocardiographic findings. Cardiac tomography assessment of the degree of valve calcification provides important additional information (28). Nonetheless, in view of the poor prognosis with medical treatment, TAVR should be considered an option in certain patients with low-gradient AS. Therefore, it is very important to know parameters that allow the identification of patients who may derive benefit from the intervention. In our study, the patients with low-gradient AS who underwent TAVR had had diagnostic confirmation of the severity of the stenosis, in some cases after dobutamine stress echo, or after considering a high degree of valve calcification on tomography. Nonetheless, in these patients, valve calcification had no predictive value for functional improvement after TAVR.

Remarkably, the developed GAPA score showed a notable discriminatory value in the low-gradient population regardless of stroke volume. In this group, the score works independently with respect to the gradient, through the other three variables that make up the score.

Prospective validation studies for this novel score, in larger cohorts of patients undergoing TAVR, should be warranted.

### Potential clinical implications of this predictive model

The use of this predictive tool would be relevant in two aspects: an indication of TAVR and tailoring of post-TAVR treatment.

On the indication aspect, the use of this score could be useful in the decision-making process in certain cases, particularly, when the indication for TAVR is doubtful, due to a borderline risk of futility due to a certain degree of frailty or the presence of relevant comorbidities associated with worse outcomes, such as chronic obstructive pulmonary disease, advanced renal failure, and some oncologic conditions. In addition, there are cases showing low-flow/low-gradient AS with inconclusive categorization of AS severe, even after evaluation of calcification with CT. In such cases, this score adds valuable information to consider. Finally, patients presenting with an unclear symptomatic status are associated with moderate-severe aortic stenosis. The score could identify those patients more likely to improve after the procedure.

In the setting of the patient already treated with TAVR, there are studies suggesting a benefit of renin–angiotensin system inhibition in these patients undergoing TAVR for aortic stenosis, or at least in a notable proportion of them (29, 30). It could also be important to implement other measures in patients identified as being at greater risk of not improving, such as stricter control of BP, weight, and, very importantly, physical rehabilitation activities (31, 32).

### Limitations

As with any study, there are limitations to our study. A high number of measurements were made sequentially over time and aligned with functional status and yet is not a large cohort, and findings should be validated in a larger population of patients currently treated with TAVR.

The definition of objective functional improvement was specific to the study and, although well thought out, may be questionable. However, it was sufficiently precise and at the same time conservative, as was confirmed by observing how the group considered without improvement showed even worse post-TAVR performance in the walking test and the absence of changes in heart failure biomarkers. In addition, the classification also showed important prognostic implications.

The validity of non-invasive assessment of central pressures, pulse wave velocity and aortic pressure curves in the context of AS has been debated. Our group has shown a high correlation of these metrics with invasive measurements in patients with AS, although insufficient to accurately estimate values at the individual level according to certain criteria (9). In any case, the behavior of the estimated pressures and the augmentation index, from pre- to post-TAVR, was very similar to that reported in invasive studies (20), and, as the results of our analysis show, the augmentation index is clearly related to the functional outcome of patients regardless of the valvular gradient.

The assessment time of 6 months can be discussed; however, we know from previous studies that the improvement after TAVR is rapid, being evident even at 30 days (25, 26). On the contrary, a later evaluation, especially in an elderly population such as this one, may be affected by the concurrence or progression of other unrelated pathological processes, such as coronary artery disease or certain comorbidities.

## Conclusion

In this contemporary cohort of patients undergoing TAVR, 20% of patients experience no objective functional improvement, compared to 12% who subjectively deny such improvement. This lack of objective improvement had important effects on 2-year survival. A series of baseline non-invasively measured variables have been identified that independently predict such improvement and that allowed the design of a score whose performance was good in the global and in the low-gradient populations of derivation and validation cohorts.

## Data availability statement

The raw data supporting the conclusions of this article will be made available by the authors, without undue reservation.

## Ethics statement

The studies involving human participants were reviewed and approved by IDIVAL Comite de Ensayos Clinicos de Cantabria. The patients/participants provided their written informed consent to participate in this study.

## Author contributions

JT, GV, EB-A, and EE: research idea and study design. GV, FS, D-HL, CR, PL, JC, MB, VF-S, MG-U, AG, and SB: data acquisition. JT, GV, EB-A, TG, JB, and EE: data analysis/interpretation. JT, GV, EB-A, and TG: statistical analysis. JZ, JV, and EE: supervision and mentorship. All authors have contributed important intellectual content during manuscript drafting or revision, accepts accountability for the overall work by ensuring that questions pertaining to the accuracy or integrity of any portion of the work are appropriately investigated and resolved, and contributed to the drafting of the manuscript review and approved the final version.

## Supplementary material

Supplementary Figure 1 Protocol for the workflow of the study. TAVR, Transcatheter Aortic Valve Replacement; TT, transthoracic.

Supplementary Figure 2 Physiological and anatomical variables analyzed in the cardiovascular system. CT, cardiac tomography; LVOT, left ventricular outflow tract; SVi, stroke volume index; Zva, valvuloarterial impedance.

Supplementary Figure 3 Flow chart of the study.

Supplementary Figure 4 Receiver operating curves for the Score GAPA (mean gradient, augmentation index_75_, posterior wall thickness, atrial fibrillation) predictive of functional improvement after TAVR in the general population with aortic stenosis, in the low-gradient (mean gradient < 40 mmHg), and low-gradient/low-flow (stroke volume index < 35 ml/m^2^) aortic stenosis subpopulations.

Supplementary Figure 5 Plotting baseline values of mean aortic valve gradient against augmentation index_75_ and proportion of objective functional improvement per quadrant based on best cutoff values.
