# Supplementary figures and images for: A new integrative approach to assess aortic stenosis burden and predict objective functional improvement after TAVR
Source: Front Cardiovasc Med. 2023 Mar 2;10:1118409. doi: 10.3389/fcvm.2023.1118409 (PMC10017439; doi:10.3389/fcvm.2023.1118409)

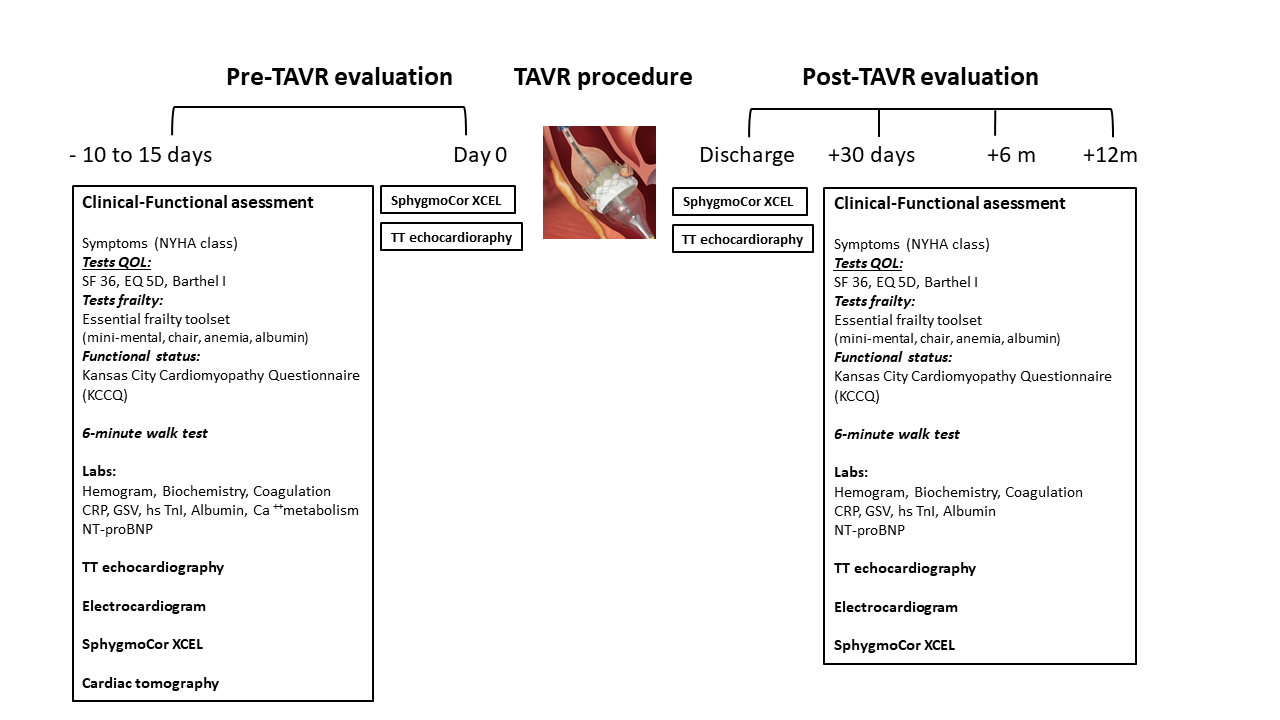

Supplement: Supplementary Figure 1 — Protocol for the workflow of the study. TAVR, Transcatheter Aortic Valve Replacement; TT, transthoracic. [file Image_1.TIF]

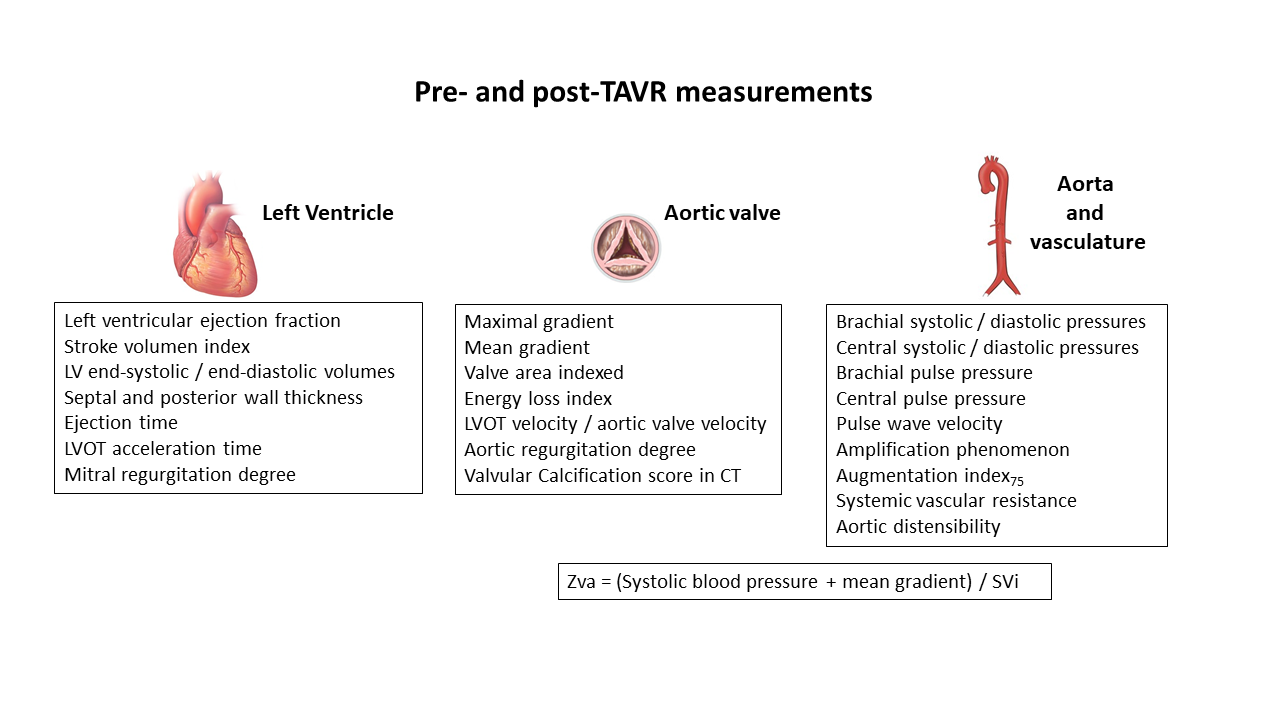

Supplement: Supplementary Figure 2 — Physiological and anatomical variables analyzed in the cardiovascular system. CT, cardiac tomography; LVOT, left ventricular outflow tract; SVi, stroke volume index; Zva, valvuloarterial impedance. [file Image_2.TIF]

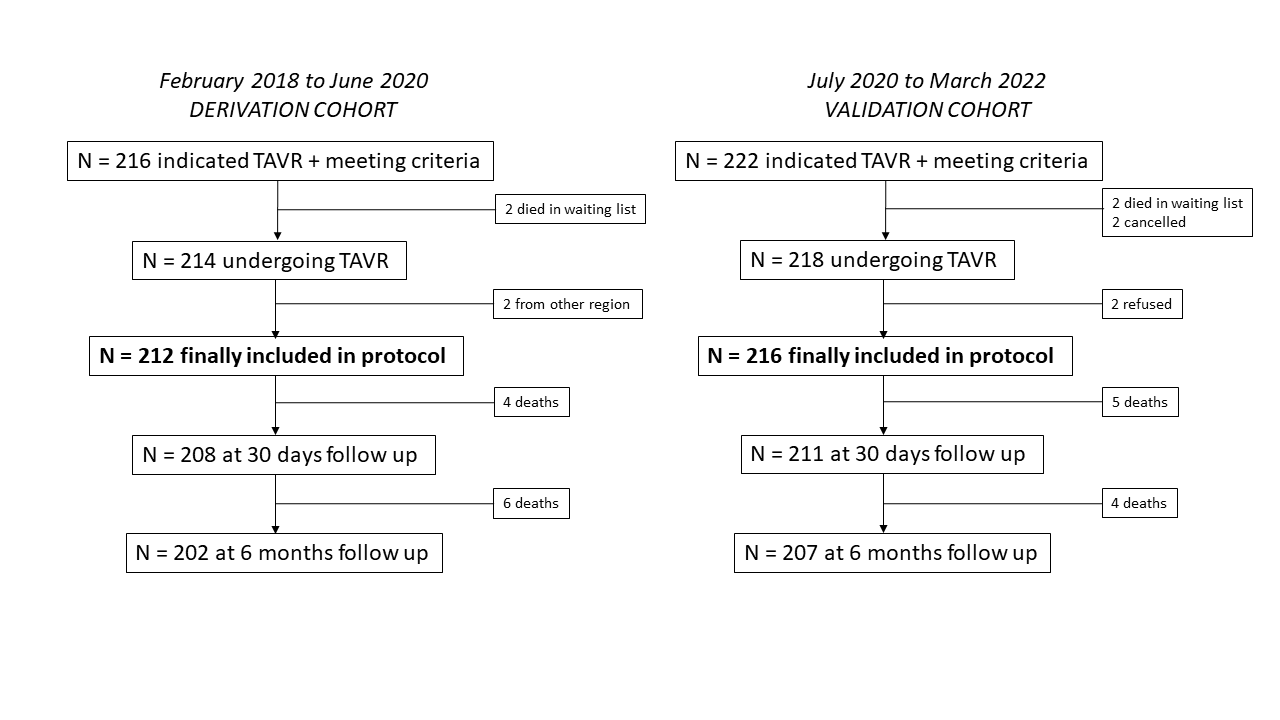

Supplement: Supplementary Figure 3 — Flow chart of the study. [file Image_3.TIF]

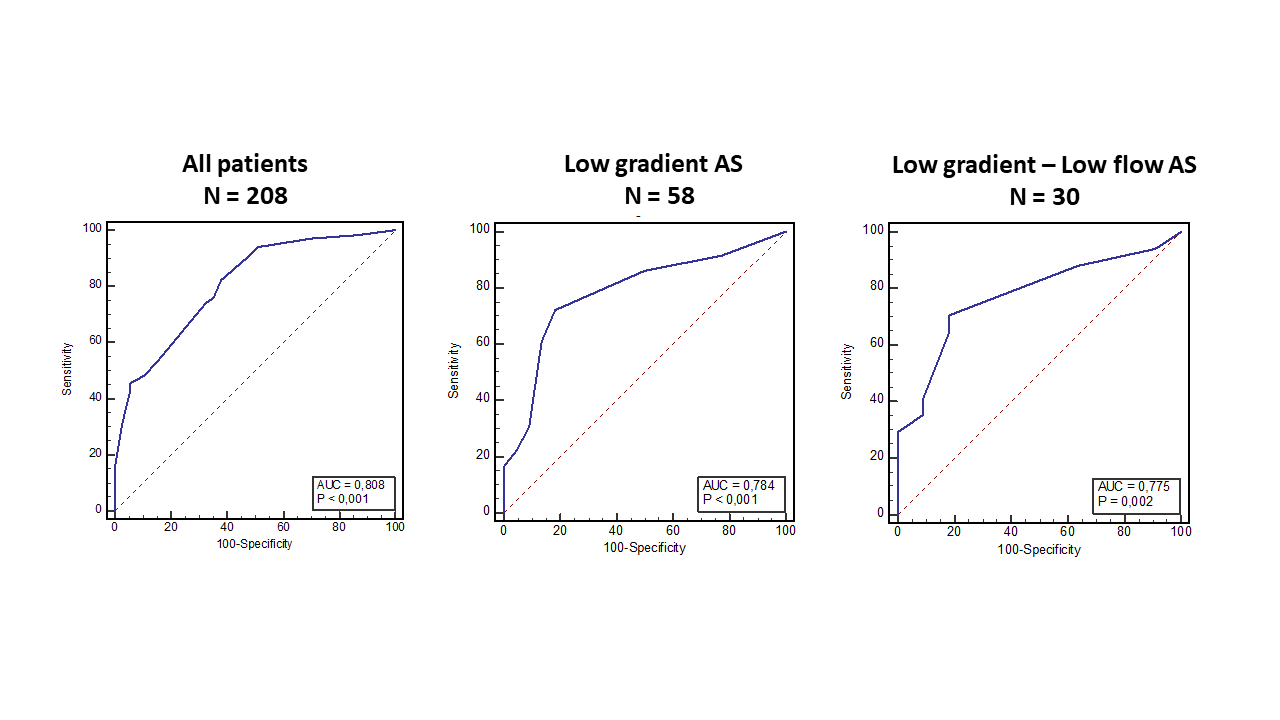

Supplement: Supplementary Figure 4 — Receiver operating curves for the Score GAPA (mean gradient, augmentation index75, posterior wall thickness, atrial fibrillation) predictive of functional improvement after TAVR in the general population with aortic stenosis, in the low-gradient (mean gradient < 40 mmHg), and low-gradient/low-flow (stroke volume index < 35 ml/m2) aortic stenosis subpopulations. [file Image_4.TIF]

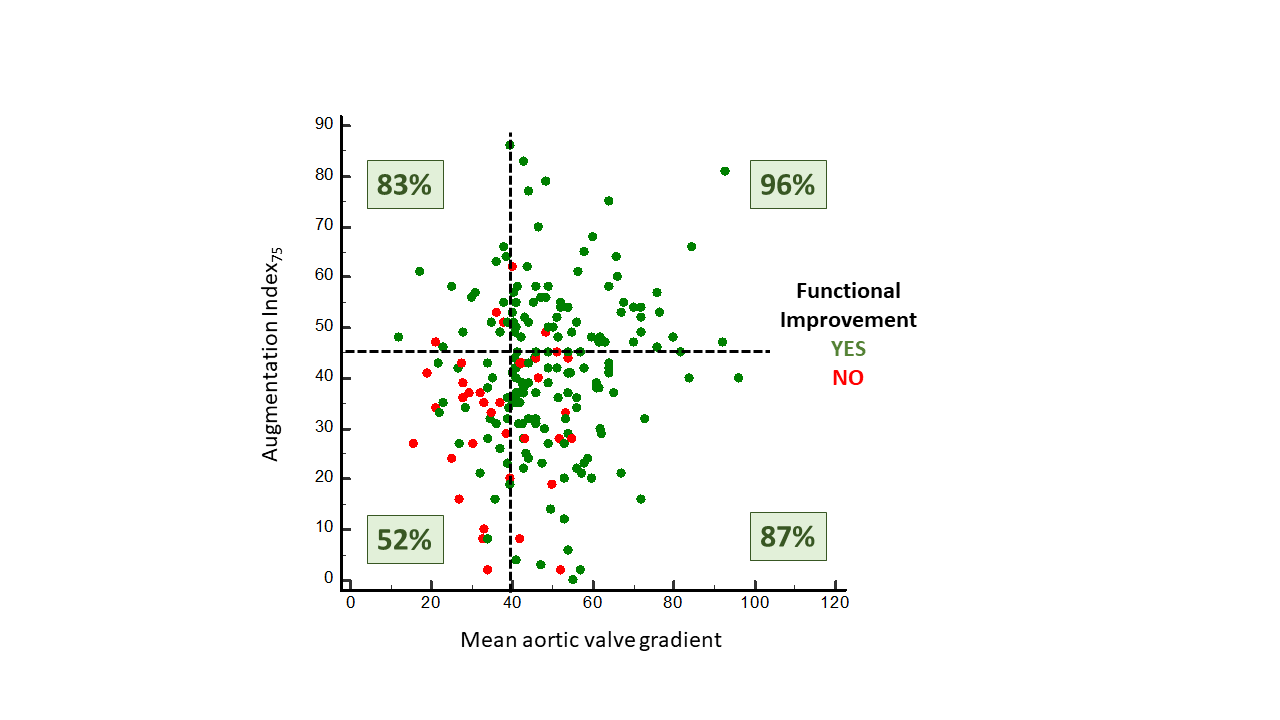

Supplement: Supplementary Figure 5 — Plotting baseline values of mean aortic valve gradient against augmentation index75 and proportion of objective functional improvement per quadrant based on best cutoff values. [file Image_5.TIF]
