# Supplementary material for: A new integrative approach to assess aortic stenosis burden and predict objective functional improvement after TAVR
Source: Front Cardiovasc Med. 2023 Mar 2;10:1118409. doi: 10.3389/fcvm.2023.1118409 (PMC10017439; doi:10.3389/fcvm.2023.1118409)
Supplement: Supplementary file 6 [file Table_1.DOCX]

**Suppl Table 1. Baseline and procedural characteristics**

| **Baseline characteristics (N = 212)** | | | |
| --- | --- | --- | --- |
| Age,years | 82 (77-85) | Mitral valve disease | 35 (16.5%) |
| Female gender | 118 (55.7%) | Atrial fibrillation | 78 (36.8%) |
| Diabetes | 66 (31.1%) | Previous pacemaker | 10 (4.7%) |
| High blood pressure | 177 (83.5%) | Chronic renal failure  GFR < 60 ml/min  GFR < 30 ml/min | 93 (44%)  11 (5.2%) |
| Dyslipidemia | 156 (73.6%) | Pulmonary disease | 34 (16%) |
| Coronary artery disease | 66 (31%) | Liver disease | 8 (3.8%) |
| Previous MI | 18 (8.5%) | History of cancer | 40 (19%) |
| Previous PCI  < 6 months | 42 (19.8%)  22 (10.4%) | Agatston calcium score | 3127 ± 1637 |
| Previous CABG | 4 (1.9%) | EuroSCORE II | 3.3 ± 2.6 |
| Carotid disease | 5 (2.4%) | STS-score mortality | 3.4 ± 2.1 |
| Peripheral vascular disease | 18 (8.5%) | Mitral valve disease | 35 (16.5%) |
| Age,years | 82 (77-85) | Atrial fibrillation | 78 (36.8%) |
| **Symptomatic and functional status** | | **Procedural characteristics and complications** | |
| NYHA  Class I  Class II  Class III  Class IV | 0  139 (65.6%)  67 (31.6%)  6 (2.8%) | Size of valve prothesis  23mm  26mm  29mm | 83 (39.2%)  106 (50%)  23 (10.8%) |
| KCCQ | 59 (50-69) | Balloon expandable valve prosthesis | 212 (100%) |
| Test SF-36 | 45.1 ± 17.8 | Femoral TAVR Access site | 212 (100%) |
| Test EQ-5D | 54.9 ± 14.8 | Predilatation | 113 (53.3%) |
| Barthel index | 94 ± 13 | Postdilatation | 10 (4.7%) |
| Charlson comorbility index | 5.6 ± 2.2 | Coronary obstruction | 3 (1.4%) |
| Essential Frailty Toolset  0  1-2  3-4  5 | 71 (33.5%)  111 (52.4%)  30 (14.1%)  0 | Valve embolization  Second valve implantation  Aortic annulus rupture  Stroke  Major vascular complications | 0  0  3 (1.4%)  1 (0.5%)  7 (3.3%) |

Values are n (%), mean ± SD, or median (25th-75th interquartile range), depending on variable distribution.

MI: Myocardial infarction; PCI: Percutaneous coronary intervention; CABG: Coronary artery bypass graft; GFR: Glomerular filtration rate; STS-score mortality: Society of Thoracic Surgeons score of mortality; NYHA: New York Heart Asocciation; KCCQ: Kansas City Cardiomiopathy Questionnarie; SF-36: The Short Form-36 Health Survey; EQ-5D: European Quality of life 5 Dimensions
