# Supplementary material for: A new integrative approach to assess aortic stenosis burden and predict objective functional improvement after TAVR
Source: Front Cardiovasc Med. 2023 Mar 2;10:1118409. doi: 10.3389/fcvm.2023.1118409 (PMC10017439; doi:10.3389/fcvm.2023.1118409)
Supplement: Supplementary file 7 [file Table_2.DOCX]

**Suppl Table 2: Physiological and anatomical variables at baseline and at 6 months follow-up post-TAVR.**

|  | **Pre-TAVR**  **N = 208** | **Post-TAVR**  **N = 208** | **P value** |
| --- | --- | --- | --- |
| Left ventricular ejection fraction (%) | 60 (50-67) | 63 (53-70) | 0.03 |
| Stroke volume index (ml/m2) | 44 (35.4-53.1) | 47.6 (39-57.6) | 0.001 |
| IV septum diastolic thickness (mm) | 14.2 ± 2.6 | 13.5 ± 2.3 | 0.003 |
| Posterior wall diastolic thickness (mm) | 12.8 ± 2.4 | 12.3 ± 2.2 | 0.02 |
| Maximal aortic gradient (mmHg) | 77 (65.5-92.1) | 21 (17-28.5) | 0.0001 |
| Mean aortic gradient (mmHg) | 46 (39-56) | 11 (8.7-14) | 0.0001 |
| Aortic valve área (cm^2^) | 0.7 (0.58-0.88) | 1.9 (1.5-2.3) | 0.0001 |
| Indexed aortic valve area (cm^2^/m^2^) | 0.4 (0.33-0.5) | 1 (0.9-1.3) | 0.0001 |
| Energy loss index (cm^2^/m^2^) | 0.45 (0.35-0.56) | 1.5 (1.1-2) | 0.0001 |
| LVOT velocity/Aortic valve velocity | 0.21 (0.17-0.25) | 0.5 (0.42-0.6) | 0.0001 |
| Sphygmocor XCEL  Central SBP (mmHg)  Central DBP (mmHg)  Central PP  Pulse wave velocity (m/s)  Amplification phenomenon  Augmentation index_75_ | 130.9 ± 19.5  72 (65-82)  56.9 ± 17.4  11.6 ± 3.2  24 (17-31)  40 ± 16.3 | 138.6 ± 19.3  80 (71-87)  58.9 ± 15.4  13.4 ± 3.4  19 (13-28)  29.5 ± 12.7 | 0.0001  0.0001  0.09  0.0001  0.0001  0.0001 |
| Systemic vascular resistance (dyna.seg.cm^-5^) | 1372 (1051-1735) | 1380 (1060-1704) | 0.78 |
| Aortic distensibility (cm2 dyna^-1^ 10 ^-6^)  TTE  TEE | 1.2 ± 0.9  1.1 ± 1 | 1 ± 0.9  1 ± 0.96 | 0.08  0.53 |
| Zva (mmHg.ml^-1^.m^-2^) | 4.13 (3.4-5.2) | 3.2 (2.6-4) | 0.0001 |
| **Functional assesment** | | | |
| NT-proBNP (pg/ml) | 1750 (705-3886) | 609 (244-1506) | 0.0001 |
| 6 minute walk test (m) | 247.5 ± 106.8 | 274.9 ± 111.5 | 0.001 |
| KCCQ | 60.4 ± 13.5 | 78.6 ± 13.5 | 0.0001 |
| NYHA  I  II  III  IV | 0  139 (65.6%)  67 (31.6%)  6 (2.8%) | 160 (76.9%)  46 (22.1%)  2 (1%)  0 | 0.0001 |

Values are n (%), mean ± SD, or median (25th-75th interquartile range), depending on variable distribution.

TAVR: Transcatheter aortic valve replacement; SVi: stroke volumen indexed; LVOT: Left ventricular outflow tract; SBP: Systolic blood pressure; DBP: Diastolic blood pressure; PP: Pulse pressure; TTE: Transthoracic echocardiogram; TEE: Transesophageal echocardiogram; Zva: Valvuloarterial impedance; NT-proBNP: N-terminal-pro hormone brain natriuretic peptide; KCCQ: Kansas City Cardiomiopathy Questionnarie ; NYHA: New York Heart Association.
