# Supplementary material for: A new integrative approach to assess aortic stenosis burden and predict objective functional improvement after TAVR
Source: Front Cardiovasc Med. 2023 Mar 2;10:1118409. doi: 10.3389/fcvm.2023.1118409 (PMC10017439; doi:10.3389/fcvm.2023.1118409)
Supplement: Supplementary file 8 [file Table_3.DOCX]

**Suppl Table 3. Clinical outcomes at 2 years follow up after discharge**

|  | **Discharge-30 days** | **1-6 m** | **6-12 m** | **12-24 m** | **Overall** |
| --- | --- | --- | --- | --- | --- |
|  | **N = 208** | | | | |
| Death | 0 | 5 | 10 | 12 | 27 (13%) |
| Stroke  Ischemic  Hemorrhagic | 0  0  0 | 5  4  1 | 2  2  0 | 6  5  1 | 13 (6.2%)  11 (5.3%)  2 (1%) |
| Myocardial infarction | 0 | 0 | 1 | 4 | 5 (2.4%) |
| Percutaneous coronary intervention | 0 | 0 | 0 | 2 | 2 (1%) |
| Heart failure admission | 4 | 15 | 7 | 9 | 35 (17%) |
| New pacemaker | 2 | 3 | 1 | 4 | 10 (4.8%) |
| Aortic valve replacement | 0 | 1 | 0 | 0 | 1 (0.5%) |
| Major Vascular complications | 0 | 0 | 0 | 0 | 0 |
| Major Bleeding | 0 | 2 | 2 | 6 | 10 (4.8%) |
| Any admission | 11 | 34 | 22 | 33 | 100 (48%) |
