# Supplementary material for: A new integrative approach to assess aortic stenosis burden and predict objective functional improvement after TAVR
Source: Front Cardiovasc Med. 2023 Mar 2;10:1118409. doi: 10.3389/fcvm.2023.1118409 (PMC10017439; doi:10.3389/fcvm.2023.1118409)
Supplement: Supplementary file 9 [file Table_4.DOCX]

**Suppl Table 4. Performance of different parameters as compared with the GAPA score for prediction of objective functional improvement after TAVR.**

**Overall AS Low-gradient AS**

**AUC p AUC p**

GAPA Score 0.81 0.001 0.78 0.001

Mean aortic gradient 0.68 0.001 0.59 0.23

Augmentation Index_75_ 0.66 0.001 0.62 0.10

Valvulo-arterial impedance 0.57 0.26 0.56 0.42

Stroke volume index 0.56 0.24 0.52 0.73

Energy loss index 0.57 0.25 0.56 0.41

AUC: area under the receiver operating characteristic curve; GAPA Score (mean Gradient, Augmentation index_75_, Posterior wall thickness, Atrial fibrillation)
