# Supplementary material for: A new integrative approach to assess aortic stenosis burden and predict objective functional improvement after TAVR
Source: Front Cardiovasc Med. 2023 Mar 2;10:1118409. doi: 10.3389/fcvm.2023.1118409 (PMC10017439; doi:10.3389/fcvm.2023.1118409)
Supplement: Supplementary file 10 [file Table_5.DOCX]

**Suppl Table 5. Clinical outcomes at 2 years follow up after discharge according to objective functional improvement**

|  | **Objective funtional improvement**  **N=169** | **Not objective funtional improvement**  **N=39** | **P value** |
| --- | --- | --- | --- |
| Death | 15 (8.9%) | 12 (30.8%) | 0.0001 |
| Stroke  Ischemic  Hemorrhagic | 10 (6%)  9 (5.4%)  1 (0.6%) | 3 (7.7%)  2 (5.1%)  1 (2.6%) | 0.7 |
| Myocardial infarction | 4 (2.4%) | 1 (2.6%) | 0.9 |
| Percutaneous coronary intervention | 1 (0.6%) | 1 (2.6%) | 0.34 |
| Heart failure admission | 21 (12.4%) | 14 (36%) | 0.0001 |
| New pacemaker | 7 (4.1%) | 3 (7.7%) | 0.35 |
| Aortic valve replacement | 1 (0.6%) | 0 | 1 |
| Major Vascular complications | 0 | 0 |  |
| Major Bleeding events | 9 (5.3%) | 1 (2.6%) | 0.48 |
| Any hospital readmission | 77 (45.6%) | 23 (59%) | 0.09 |
