# Supplementary material for: A new integrative approach to assess aortic stenosis burden and predict objective functional improvement after TAVR
Source: Front Cardiovasc Med. 2023 Mar 2;10:1118409. doi: 10.3389/fcvm.2023.1118409 (PMC10017439; doi:10.3389/fcvm.2023.1118409)
Supplement: Supplementary file 11 [file Table_6.DOCX]

**Supplementary Table 6.** Based on objective and subjective functional improvement four subgroups were identified presenting the following 2-years death rates.

**FUNCTIONAL IMPROVEMENT N Death at 2 years**

Objective + Subjective + 161 14 (8.7%)

Objective + Subjective - 8 1 (12.5%)

Objective - Subjective + 26 6 (23%)

Objective - Subjective - 13 6 (46%)
