# Supplementary material for: A new integrative approach to assess aortic stenosis burden and predict objective functional improvement after TAVR
Source: Front Cardiovasc Med. 2023 Mar 2;10:1118409. doi: 10.3389/fcvm.2023.1118409 (PMC10017439; doi:10.3389/fcvm.2023.1118409)
Supplement: Supplementary file 12 [file Table_7.DOCX]

**Suppl Table 7. Baseline clinical characteristics in validation cohort**

|  | **N = 216** |
| --- | --- |
| Age,years | 80 (74-84) |
| Female gender | 116 (53.7%) |
| Diabetes | 60 (27.7%) |
| High blood pressure | 185 (85.6%) |
| Dyslipidemia | 151 (70%) |
| Coronary artery disease | 72 (33.3%) |
| Previous MI | 18 (8.3%) |
| Previous PCI  < 6 months | 43 (20%)  17 (7.8%) |
| Previous CABG | 3 (1.4%) |
| Carotid disease | 7 (3.2%) |
| Peripheral vascular disease | 20 (9.2%) |
| Mitral valve disease | 30 (13.8%) |
| Atrial fibrillation | 71 (32.8%) |
| Previous pacemaker | 7 (3.2%) |
| Chronic renal failure  GFR < 60 ml/min  GFR < 30 ml/min | 101 (46.7%)  8 (3.7%) |
| Pulmonary disease | 35 (16.2%) |
| Liver disease | 3 (1.4%) |
| History of cancer | 38 (17.6%) |
| Agatston calcium score | 2990 ± 1267 |
| EuroSCORE II | 3.1 ± 2.5 |
| STS-score mortality | 3 ± 1.9 |
| **Symptomatic and functional status** | |
| NYHA  Class I  Class II  Class III  Class IV | 0  149 (69%)  60 (27.7%)  7 (3.2%) |
| KCCQ | 54 (50-68) |
| Test SF-36 | 43.1 ± 16.8 |
| Test EQ-5D | 54 ± 14 |
| Barthel index | 90 ± 13 |
| Charlson comorbility index | 5.1 ± 2.2 |
| Essential Frailty Toolset  0  1-2  3-4  5 | 86 (40%)  111 (51.4%)  19 (8.8%)  0 |

Values are n (%), mean ± SD, or median (25th-75th interquartile range), depending on variable distribution.

MI: Myocardial infarction; PCI: Percutaneous coronary intervention; CABG: Coronary artery bypass graft; GFR: Glomerular filtration rate; STS-score mortality: Society of Thoracic Surgeons score of mortality; NYHA: New York Heart Asocciation; KCCQ: Kansas City Cardiomiopathy Questionnarie; SF-36: The Short Form-36 Health Survey; EQ-5D: European Quality of life 5 Dimensions
