# Supplementary material for: A new integrative approach to assess aortic stenosis burden and predict objective functional improvement after TAVR
Source: Front Cardiovasc Med. 2023 Mar 2;10:1118409. doi: 10.3389/fcvm.2023.1118409 (PMC10017439; doi:10.3389/fcvm.2023.1118409)
Supplement: Supplementary file 13 [file Table_8.DOCX]

|  | **N = 216** |
| --- | --- |
| Balloon expandable valve prosthesis | 216 (100%) |
| Size of valve prothesis  23mm  26mm  29mm | 80 (37%)  109 (50.4%)  27 (12.5%) |
| Femoral TAVR Access site | 216 (100%) |
| Predilatation | 71 (32.8%) |
| Postdilatation | 8 (3.7%) |
| Coronary obstruction | 2 (0.9%) |
| Valve embolization | 0 |
| Second valve implantation | 0 |
| Aortic annulus rupture | 1 (0.5%) |
| Stroke | 3 (1.4%) |
| Major vascular complications | 5 (2.3%) |

**Suppl Table 8. Procedural characteristics in validation cohort**
